# Perceptual and Biochemical Responses in Relation to Different Match-Day +2 Training Interventions in Soccer Players

**DOI:** 10.3389/fphys.2021.685804

**Published:** 2021-06-24

**Authors:** Athos Trecroci, Enrico Perri, Giovanni Lombardi, Giuseppe Banfi, Riccardo Del Vescovo, Ermes M. Rosa, Giampietro Alberti, F. Marcello Iaia

**Affiliations:** ^1^Department of Biomedical Sciences for Health, Università degli Studi di Milano, Milan, Italy; ^2^Laboratory of Experimental Biochemistry and Molecular Biology, IRCCS Istituto Ortopedico Galeazzi, Milan, Italy; ^3^Department of Athletics, Strength and Conditioning, Poznań University of Physical Education, Poznań, Poland; ^4^Vita-Salute San Raffaele University, Milan, Italy; ^5^Villa Stuart Sport Clinic, Rome, Italy; ^6^Marathon Sports Medical Center, Brescia, Italy

**Keywords:** football (soccer), fatigue, active recovery, congested schedule, physiology

## Abstract

The aim of this study was to examine the impact of two different post-match training interventions on the subsequent recovery of perceptual and biochemical parameters after the game. In a crossover design, eight sub-elite players underwent a soccer-specific training (SST) and an active recovery (AR) regimen on the second day after a match (+48 h). Muscle soreness as well as muscle damage (creatine kinase, CK), inflammatory (C-reactive protein and interleukin 6), immunological (e.g., lymphocytes, neutrophils, and monocytes), and endocrine (cortisol) markers were obtained at baseline (−72 h), immediately after (0 h), and 72 h post-match (+72 h). AR promoted a higher restoration of muscle soreness values (*P* = 0.004, η^2^_*p*_ = 0.49) together with a better restoration of CK within 72 h post-match compared with SST (*P* = 0.04, η^2^_*p*_ = 0.36). Conversely, no significant (*P* > 0.05, η^2^_*p*_ < 0.91) differences were observed in the recovery timeframe of inflammatory, immunological, and endocrine responses between SST and AR. Overall, AR elicited a quicker muscle soreness and CK restoration compared to SST intervention at 72 h post-match. Such information provides novel evidence-based findings on the appropriateness of different recovery strategies and may aid to improve the practitioners’ decision-making process when two consecutive games are played within 3 days.

## Introduction

During the competitive soccer season, players often undergo congested fixture schedules where they are usually required to play multiple games within a very short period of time. This imposes pronounced biomechanical stress, which may consequently prevent players from optimally recovering during the following days. This condition can induce a prolonged fatigue status, which refers to a failure in maintaining the required task that was achievable within the pre-match time frame (Pyne and Martin, 2011). Indeed, players’ fatigue status may be prolonged for several hours/days after a single match, causing performance reductions (e.g., sprint ability) (Dupont et al., 2010; Silva et al., 2018), neuromuscular impairments (e.g., maximal voluntary contraction) (Krustrup et al., 2011; Draganidis et al., 2015; Silva et al., 2018; Trecroci et al., 2020), as well as perceptual discomfort (e.g., muscle soreness) and biochemical perturbations (e.g., muscle damage, inflammatory and immunological markers) (Silva et al., 2018). In particular, muscle soreness and uric acid (inflammatory marker) (Sanchis-Gomar et al., 2015) did not return to baseline levels before 48 h after a match (Ascensão et al., 2008; Fatouros et al., 2010), while plasma creatine kinase (CK) activity remained significantly three- to eight-fold higher during the next 72 h (Ascensão et al., 2008; Fatouros et al., 2010), indicating a muscle damage. Additionally, neutrophil and interleukin 6 were shown to be increased up to 48 h after a game (match day + 2) (Romagnoli et al., 2016). Thus, the exacerbated muscle damage and inflammatory and immune markers observed in the hours following a soccer game may play a determinant role for the slow recovery timeframe and the subsequent players’ inability to reach optimal levels of readiness. Nonetheless, the restoration of selected biochemical parameters may also be further affected by the practices carried out in the days after the game (Ekstrand, 2004; Draganidis et al., 2015; Trecroci et al., 2020). While the vast majority of the studies have investigated the recovery strategies (Fatouros et al., 2010; Romagnoli et al., 2016), the current literature lacks solid scientific evidence regarding the most appropriate type of training [e.g., active recovery (AR) or soccer-specific training (SST) sessions] to be performed in the days following a match and its impact on subsequent restoration of physiological and performance markers.

Only few studies have examined the effect of post-game interventions on the following physiological response and exercise performance (Andersson et al., 2008, 2010) in female soccer players. Andersson et al. (2008, 2010) compared the impact of 1-h AR (non-soccer-specific session including submaximal cycling at 60% peak heart rate and resistance training at <50% one-repetition maximum) versus passive recovery on neuromuscular, biochemical (e.g., CK and uric acid), and perceptual responses during the 72-h period between two matches. The authors found that the AR failed to affect CK concentration, acid uric levels, and perceived muscle soreness. However, the ecological validity of the latter study may be questioned as the athletes’ sport-specific needs in preparation of the next match-play were not considered. Of note, in a real scenario, players are supposed to practice their technical–tactical skill between two consecutive games. To the best of our knowledge, only one investigation (Trecroci et al., 2020) researched the effects of different field-based training interventions performed 2 days after the game on the subsequent recovery of physical and neuromuscular performance. Trecroci et al. (2020) compared 1 h of soccer-specific activities simulating a pre-match training session (i.e., small-sided games, attacking/defending solutions and offensive set pieces) versus ∼30 min of AR (i.e., low-intensity technical drills and straight-line jogging). It was demonstrated that low-intensity AR promoted a better restitution of knee flexor muscle force production in the post-game period (Trecroci et al., 2020). However, although novel information on the recovery kinetics of targeted physical and neuromuscular components was provided, the effect of different strategies on the time course of specific biochemical and perceptual parameters remains unknown. In particular, whether an augmented blood flow induced by high-intensity exercise may contribute to enhance the restoration of specific immunological and inflammatory markers is still unclear.

A better understanding on how and to which extent such variables could be affected by different field-based training interventions would provide new insight into the selection of the most appropriate training practices for emphasizing the recovery processes and maximizing players’ readiness. This may have important implications when two or more games are separated by only few hours (i.e., 72 h). Thus, the aim of the present study was to assess the recovery kinetics of selected perceptual and biochemical parameters 72 h after a soccer match in relation to different types of match-day +2 training interventions.

## Materials and Methods

### Participants

Nine young male sub-elite soccer players (age 17.7 ± 0.55 years, 177 ± 2.3 cm, body mass 65.85 ± 6.0 kg) who have been playing for a minimum of 8 years volunteered to participate in the study. All players were part of the same team competing in the U19 National League. Exclusion criteria were as follows: (i) history of febrile illness and lower-limb injuries in the 8 weeks prior to the study; and (ii) a compliancy of less than four training sessions and a game per week during the 7 days before the experimental period. Players, parents, or legal guardians were deeply informed about the research purpose and any potential risks of the experiment before given a written informed consent to participate. If under the age of 18, the player and his parents or legal guardian signed the written informed consent. The study was approved by the Ethical Committee of the Università degli Studi di Milano (32/16 approval number) in accordance with the Helsinki declaration.

### Experimental Design

**FIGURE 1:**
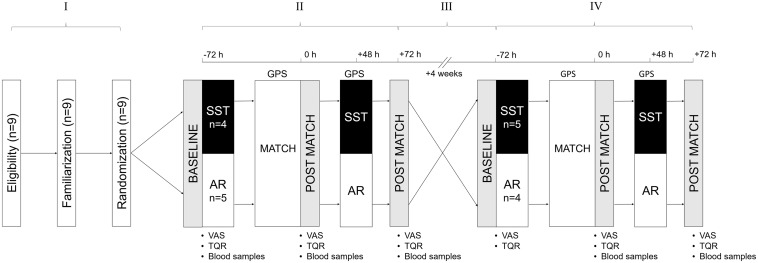
A schematic representation of the experimental schedule. The group-specific training interventions were completed 48 h after the match, while VAS scores and blood samples were collected before (−72 h), immediately (0 h), and after (72 h) the match. GPS, global positioning system; SST, sport-specific training session; AR, active recovery session; VAS, visual analog scale; TQR, total quality of recovery.

A crossover design was utilized to study the effects of two different training regimes carried out 48 h after the game on the recovery of post-match perceptual and biochemical variables measured 72 h after the game. The entire protocol was conducted during the in-season period and consisted of (I) initial procedures including a familiarization period, (II) a first experimental phase, (III) a 4-week washout period, and (IV) a second experimental phase (Figure 1). During both experimental phases, all players were monitored and tested on three different time points: 72 h before the match (baseline), immediately after the match (0 h), and 72 h post-match (+72 h). On the second day after the match (+48 h), the participants performed either an AR or a SST session based on different durations (30 min vs. 1 h) and intensity (low vs. high).

Each testing session included a blood sample collection as well as perceptual assessment (self-reported values of muscle soreness and quality of recovery). The match played consisted of a 90-min friendly game with no substitutions against a team of the same competitive level, and it was preceded by a 15-min standardized warmup. Height and body mass were obtained 2 days prior to the match using a stadiometer (SECA 213, Germany) and a portable scale (SECA 813, Germany) to the nearest 1.0 cm and 0.1 kg, respectively. After 4 weeks, the participants swapped the group-specific interventions and completed the second experimental phase undergoing the same timeline and procedures as described above (Figure 1). The washout period served to minimize the influence of fatigue-induced carryover effects experienced by the players within the in-season schedule (Trecroci et al., 2020). As prior to the study, during the washout period, all players followed their in-season macrocycle training routine consisting of four ∼2-h sessions and a game per week. The weekly training content of each session was equally distributed throughout the washout period. The players did not experience any musculoskeletal issues, injury events, or diseases throughout both the experimental and washout periods.

#### Training Interventions

The day after the match, all participants were requested not to practice or to do any low-to-high physical activity or manual therapy, massages, or similar recovery strategies. On the second day after the game (+48 h), the participants performed either a SST or an AR session. Briefly, the STT lasted ∼60 min and consisted of warmup, small-sided games, and tactical drills, whereas the AR was constituted by ∼30-min light activities including low-intensity technical drills, dynamic stretching, and straight-line jogging (Trecroci et al., 2020). Overall, the experimental schedule (a resting session 24 h post-match and a training intervention 48 h post-match) followed the common post-match program (Impellizzeri et al., 2004).

#### Match-Play and Training Activities

Total and high speed run – i.e., >18 km/h – distances, estimated metabolic power (Osgnach et al., 2010), and distance covered at accelerating and decelerating (Gaudino et al., 2014) were monitored during the game and training sessions by a portable non-differential 10-Hz (standard error of measurement 5.1%, coefficient of variation <5%) (Kelly et al., 2014) global positioning system (GPS) integrated with a 400-Hz 3-D accelerometer, a 3-D gyroscope, a 3-D digital compass, and a 10-Hz 3-D magnetometer (Playertek GPS System; Kodaplay Ltd., Dundalk, Ireland). All GPS pods were turned on 15 min before each experimental session to favor an optimal acquisition of satellite signals. Moreover, each player used the same pod throughout the experimental period to avoid interunit error. Cardiovascular load [heart rate (HR)] by a dedicated belt connected to the GPS pod *via* Bluetooth signal (Playertek System; Kodaplay Ltd., Dundalk, Ireland) and rate of perceived exertion (RPE) by means of the Borg Category-Ratio–10 scale (CR10) were also recorded (Impellizzeri et al., 2004). The RPE was individually collected 20 min after each match and training session. All players were already familiar with the CR10 scale as routinely embedded in their weekly assessment procedure throughout the season.

#### Perception of Recovery

Perceived muscle soreness was evaluated using a 10-cm linear analog scale with labels that corresponded to “not pain” and “extreme pain” either end (Nosaka et al., 2002). This scale is a sensitive tool expressing an indirect measure of muscle damage (Saw et al., 2016; Silva et al., 2018) *via* subjective discomfort responses. Each participant marked his perceived level of pain in the thigh muscles. The soreness was recorded at baseline (−72 h), immediately after (0 h), and 72 h after (+72 h) the match to monitor perceptual discomfort linked to muscle fatigue. Total quality of recovery (TQR) encompassing a 6–20 Likert scale was also provided for obtaining the players’ subjective state of recovery (Kenttä and Hassmén, 1998). The TQR scale ranged from “very very poor recovery” (corresponding to six points) to “very very good recovery” (corresponding to 20 points). TQR values were collected 30 min before starting warmup in both training sessions as well as prior to the match. Both soreness and TQR scores were used within the analysis to detect potential differences in recovery timeframe.

#### Biochemical Assays

Blood samples were collected on the field after 10 min of rest in a sitting position by standard antecubital venipuncture in both ethylenediaminetetraacetate di-potassium salt (K2-EDTA) spray-coated tubes and SST II Advance serum tubes (BD Vacutainer^®^, Becton Dickinson & Co., Franklin Lakes, NJ, United States), at the indicated time points (i.e., −72, 0, and +72 h). After sampling, blood tubes were inverted 10 times, following the manufacturer’s instruction, allowed to clot for 30 min in the case of SST^TM^ II tubes, stored at 4°C, and transported to the laboratory in a dedicated box at controlled temperature within 3 h. Once in the lab, K2-EDTA anticoagulated samples were homogenized for 15 min and assayed for the following hematological parameters on a Xn-10 Sysmex (Sysmex Co., Kobe, Japan): white blood cell (WBC) count (10^3^ cells/ml), neutrophils (Neu, 10^3^ cells/ml), monocytes (Mo, 10^3^ cells/ml), and lymphocytes (Ly, 10^3^ cells/ml). Serum, obtained following centrifugation (1,300*g*, 10 min, 25°C) of SST^TM^ II Advance samples, was assayed for the following parameters: uric acid (UA, mg/dl), creatinine (sCr, mg/dl), creatine kinase (CK, U/L), high-sensitivity C-reactive protein (CRP, mg/dl) on an Architect c8000 (Abbott Co., Chicago, IL, United States), cortisol (μg/dl) on an i1000 Architect (Abbott), and interleukin 6 (IL-6, pg/ml) on a DSX (Dynex Technologies, Chantilly, VA, United States). Instruments were routinely checked using internal and external standard analyses.

During the experimental period, the participants continued with their ordinary nutritional habits as prior to the study. On the days of testing, they were instructed to follow a standardized meal plan that satisfied the macronutrient intake for athletes engaged in daily training (García-Rovés et al., 2014) and calculated based on the body mass of each player (Russell and Pennock, 2011). The standardized dietary intake was also kept during the washout period. The players did not consume any supplements throughout the experimental protocol.

### Statistical Analysis

According to the assumption of normality assessed by the Shapiro–Wilk’s test, paired *t*-tests were used to detect possible differences between the two matches. A two-way analysis of variance (ANOVA) with repeated measures was used to detect possible interactions (time × intervention) and significant main effects of time and intervention throughout the two (0 h and +72 h) and three time points (baseline, 0 h, and +72 h) for perceptual and biochemical variables, respectively. In case of significant interaction, Bonferroni’s adjustment was used for multiple comparisons. Partial eta squared (η^2^_*p*_) was used to estimate the magnitude of the difference for interactions, and the thresholds for small, moderate, and large effects were defined as 0.01, 0.06, and 0.14, respectively. The effect size (ES) (Cohen, 1988) of the multiple comparisons was also calculated to display the within-group differences for SST and AR. The ES was classified as trivial (ES < 0.2), small (0.2 < ES < 0.5), moderate (0.5 < ES < 0.8), and large (ES > 0.8). The coefficient of variation (CV) was also computed to explore intra-individual variability at baseline (for perception of recovery variables) and 0 h (for perception of recovery variables and biochemical markers) over the experimental phases. All analyses were performed using the IBM SPSS Statistics (v. 21, New York, NY, United States), and data are shown as mean ± standard deviation (SD) with 95% confidence intervals (CI) in squared brackets. An *α*-value = 0.05 was set as the criterion level of significance.

## Results

### Game Load

The two matches played during the first and the second experimental phase were similar in terms of total distance (9,938 ± 1,185 m vs. 9,889 ± 1,100 m), high speed run distance (729 ± 117 m vs. 781 ± 202 m), metabolic power (9.03 ± 1.17 vs. 8.86 ± 1.14 W/kg), as well as distance covered at accelerations of 1–2 m⋅s^–2^ (802.07 ± 136 m vs. 900.03 ± 193 m), 2–3 m⋅s^–2^ (194.47 ± 36 m vs. 226.31 ± 49 m), and >3 m⋅s^–2^ (41.47 ± 16 m vs. ± 49 11 m) (*P* > 0.05).

### Training Load

The detailed load variables of both SST and AR interventions are shown in Table 1. In SST, the average time spent between 75% and 85% and above 85% of HRmax was ∼13.8 and ∼4.5 min, respectively, whereas only 1.7 min between 75 and 85% HRmax was recorded for AR. Furthermore, the overall distances covered at accelerations (from 1 to 3 m⋅s^–2^) and decelerations (from −1 to −3 m⋅s^–2^) were more than fourfold higher in SST compared to AR.

**TABLE 1:** Perceived load assessed by CR10 Borg scale; kinematic, metabolic, cardiovascular, and mechanical load parameters assessed by GPS for both interventions.

Training interventions	RPE (a.u.)	Total distance (km)	Metabolic power score (W⋅kg^–1^)	Time in HR zone 75–85% HRmax (s)	Time in HR zone 86–96% HRmax (s)	Distance covered at acceleration of 1–2 m⋅s^–2^ (m)	Distance covered at acceleration of 2–3 m⋅s^–2^ (m)	Distance covered at deceleration of 1–2 m⋅s^–2^ (m)	Distance covered at deceleration of 2–3 m⋅s^–2^ (m)
SST	3.6 ± 1.1	4.1 ± 0.4	3.6 ± 0.4	827 ± 117	270 ± 320	352 ± 62	102 ± 38	420 ± 80	133 ± 42
AR	1.1 ± 0.4	1.8 ± 0.3	1.8 ± 0.3	106 ± 45	0 ± 0	93 ± 25	0 ± 0	123 ± 43	0 ± 0

*GPS, global positioning system; SST, soccer-specific training session; AR, active recovery session; RPE, rating of perceived exertion; HR, heart rate; HRmax, maximal heart rate.*

### Perceptual Response

**FIGURE 2:**
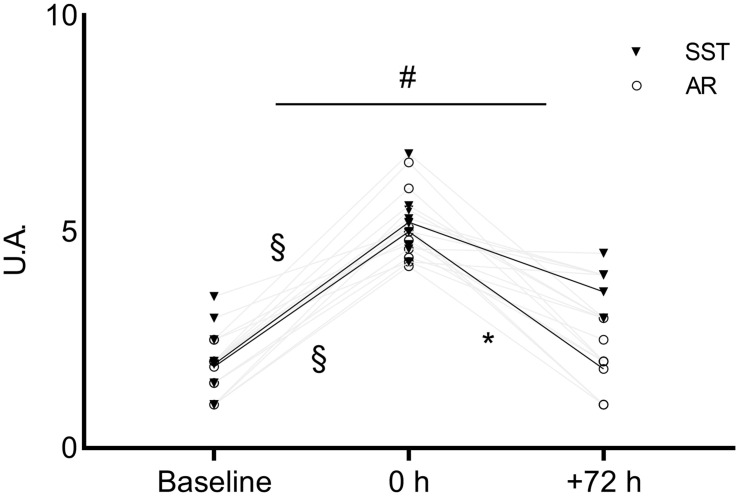
A graphical representation of the recovery pattern of muscle soreness. The bold lines identify the means of both SST and AR. ^#^Significant (*P* < 0.05) interaction throughout the time points (baseline, 0 h and +72 h). ^§^ Significant (*P* < 0.05) difference between 0 h and baseline for SST and AR. *Significant (*P* < 0.05) difference between +72 h and 0 h for AR. SST, sport-specific training session; AR, active recovery session; VAS, visual analogue scale.

A significant [*F*_(__1,16)_ = 7.901, η^2^_*p*_ = 0.497, *P* = 0.004] interaction was found in muscle soreness, which moved differently (*P* < 0.05) after the SST and AR interventions (baseline, 1.94 ± 0.91 [1.24 to 2.64 95% CI] and 1.88 ± 0.48 A.U. [1.51 to 2.26 95% CI]; 0 h, 5.22 ± 0.83 [4.66 to 5.78 95% CI] and 5.00 ± 0.82 A.U. [4.37 to 5.62 95% CI]; +72 h, 3.61 ± 0.61 [3.14 to 4.07 95% CI] and 1.83 ± 0.96 A.U. [1.29 to 2.27 95% CI]) (Figure 2). Specifically, after SST, the average soreness score from 0 to +72 h improved significantly less (*P* = 0.33, ES = 2.2) compared with AR (*P* < 0.0001, ES = 4.2). The CV was 14 and 11% at baseline and 0 h over the two experimental phases, respectively. Regarding TQR, neither significant main effects of time (*P* = 0.81) and intervention (*P* = 0.14) nor a significant interaction (*P* = 0.22) was observed between SST and AR interventions from 0 to +72 h (baseline, 16.31 ± 1.68 [15.22 to 18.01 95% CI] and 16.43 ± 1.65 A.U. [15.36 to 18.08 95% CI]; 0 h, 16.18 ± 1.51 [14.93 to 17.18 95% CI] and 16.43 ± 1.14 A.U. [15.69 to 17.53 95% CI]; +72 h, 15.43 ± 1.39 [14.53 to 16.69 95% CI] and 16.50 ± 1.1 A.U. [15.78 to 17.33 95% CI], respectively) with ESs <0.34. The CV was 0.5 and 7% at baseline and 0 h over the experimental phases, respectively.

### Biochemical Measurements

**FIGURE 3:**
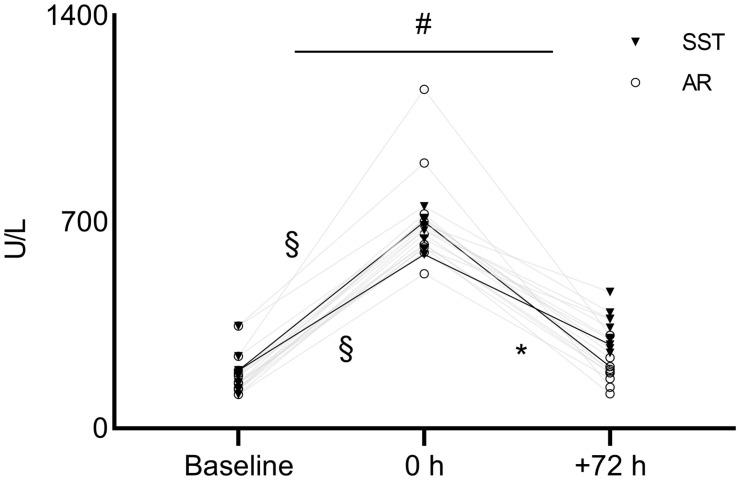
A graphical representation of the recovery pattern of CK. The bold lines identify the means of both SST and AR. ^#^Significant (*P* < 0.05) interaction throughout the time points (baseline, 0 h, and +72 h). ^§^ Significant (*P* < 0.05) difference between 0 h and baseline for SST and AR. *Significant (*P* < 0.05) difference between +72 h and 0 h for AR. SST, sport-specific training session; AR, active recovery session; CK, creatine kinase.

Regarding biochemical markers, a significant [*F*_(__1__,1__6__)_ = 4.096, η^2^_*p*_ = 0.369, *P* = 0.04] interaction was found in CK levels (Figure 3). Before both interventions, CK levels increased remarkably from baseline to 0 h in SST (from 186.12 ± 84.57 [128.2 to 239.6, 95% CI] to 570 ± 232 U/L [651 to 714, 95% CI]; *P* = 0.007, ES = 2.2) and in AR (from 186.12 ± 84.57 [128.2 to 239.6, 95% CI] to 680 ± 343 U/L [563.5 to 863, 95% CI]; *P* < 0.0001, ES = 2.0). After SST, CK levels did recover significantly less from 0 to +72 h (from 570 ± 232 to 283.87 ± 98.33 U/L [291.1 to 391.7, 95% CI]; *P* = 0.06, ES = 1.6) compared with AR (from 680 ± 343 to 209 ± 98.16 U/L [150.2 to 239, 95% CI]; *P* < 0.0001, ES = 1.9). The CV was 11% at 0 h over the two experimental phases. The absolute values of the other biochemical variables expressing muscle damage (sCr and CRP), inflammation (UA, CRP, and IL-6), endocrine (cortisol), and immunological markers (WBC, neutrophils, lymphocytes, and monocytes) are shown in Table 2 at each time point. No significant interactions were found between SST and AR interventions for all markers (*P* > 0.05). The analysis showed a significant main effect of time for all parameters (*P* < 0.005), except for CRP and lymphocytes, which did not change significantly compared throughout the time points (*P* > 0.05) (Table 2). Subsequent analyses revealed that all variables changed significantly (*P* < 0.01) from −72 to 0 h and from 0 to +72 h. *Vice versa*, all the mentioned markers did not display significant differences in the main effect of intervention (*P* > 0.05). The CV of all markers at 0 h ranged from 6 to 65% over the two experimental phases.

**TABLE 2:** Descriptive statistics (mean ± SD) for all blood markers obtained in the two treatments (SSG and AR) with *F*-values, partial eta squared (η_*p*_^2^), and *P*-values derived from the two-way ANOVA repeated measures for interaction (time × intervention) and main effects of time and intervention.

Markers	Intervention	Before Match (-72 h)	Post-Match (0 h)	Post-intervention (+72 h)	Time × Intervention			Time			Intervention		
					*F*_(2,14)_	η_*P*_^2^	*P*	*F*_(2,14)_	η_*P*_^2^	*P*	*F*_(1,7)_	η_*P*_^2^	*P*
UA (mg/dl)	SST	4.76 ± 1.03	5.93 ± 1.19*^#^	5.03 ± 1.28	1.139	0.140	0.34	18.788	0.72	** < 0.001**	2.536	0.266	0.15
	AR		5.75 ± 1.08*^#^	4.73 ± 0.96									
sCr (mg/dl)	SST	0.84 ± 0.10	1.19 ± 0.24*^#^	0.93 ± 0.19	0.700	0.91	0.51	25.505	0.78	** < 0.001**	1.702	0.196	0.23
	AR		1.12 ± 0.10*^#^	0.87 ± 0.07									
Cortisol (μg/dl)	SST	6.41 ± 1.84	14.03 ± 6.37*^#^	5.52 ± 1.29	0.506	0.067	0.61	13.746	0.66	** < 0.001**	0.512	0.068	0.49
	AR		12.12 ± 5.28*^#^	5.51 ± 1.44									
CRP (mg/dl)	SST	0.11 ± 0.10	0.20 ± 0.22	0.13 ± 0.13	2.244	0.243	0.14	0.089	0.013	0.916	3.049	0.303	0.12
	AR		0.05 ± 0.31	0.08 ± 0.09									
IL-6 (pg/ml)	SST	<1.84	3.74 ± 2.30*^#^	<1.84	0.558	0.074	0.58	5.445	0.43	**0.018**	0.558	0.074	0.47
	AR		3.06 ± 2.28*^#^	<1.84									
WBC (10^3^/ml)	SST	8.99 ± 1.65	12.11 ± 3.05*^#^	8.30 ± 1.27	0.010	0.001	0.92	39.361	0.83	** < 0.0001**	0.112	0.014	0.74
	AR		12.27 ± 2.65*^#^	8.54 ± 1.94									
Neu (10^3^/ml)	SST	5.19 ± 1.13	8.40 ± 3.05*^#^	4.55 ± 1.18	0.048	0.006	0.83	28.495	0.78	** < 0.001**	0.107	0.013	0.75
	AR		8.52 ± 2.19*^#^	4.83 ± 1.41									
Ly (10^3^/ml)	SST	2.89 ± 0.69	2.52 ± 0.55	2.82 ± 0.82	0.154	0.019	0.70	1.653	0.17	0.235	0.109	0.012	0.75
	AR		2.59 ± 0.54	2.81 ± 0.68									
Mo (10^3^/ml)	SST	0.78 ± 0.18	1.03 ± 0.14*^#^	0.73 ± 0.06	0.239	0.029	0.63	134.560	0.94	** < 0.0001**	0.012	0.002	0.91
	AR		1.01 ± 0.19*^#^	0.74 ± 0.17									

SST, sport-specific training session; AR, active recovery session; UA, uric acid; sCr, creatinine; CK, creatine kinase; CRP, C-reactive protein; IL-6, interleukin-6; WBC, white blood cells; Ly, lymphocytes; Mo, monocytes. *Significant difference toward −72 h. ^#^Significant difference toward +72 h. The bold values indicate the significance of the main effects of time.

## Discussion

The present study is the first to examine the time course of recovery of perceptual and biochemical variables after 72 h following a soccer match in response to different training strategies performed 48 h after the game (match-day +2). The most important findings were that compared to SST, AR promoted a better restoration of CK together with a higher normalization of VAS values within 72 h post-match, whereas no differences were observed in inflammatory, immunological (WBC, and Ly), and endocrine markers between the two training interventions.

Creatine kinase represents the most frequently used marker to monitor the muscle damage in several team ball sports (Doeven et al., 2018), and the results from our study may indicate that performing a SST session 2 days after a match could potentially cause prolonged muscle damage and soreness in the following day (+72 h). This is in line with recent systematic reviews showing a substantial elevation of muscle damage markers (e.g., CK) until 72 h after the game (Doeven et al., 2018; Silva et al., 2018). Besides the biochemical stress imposed by a soccer match, such a prolonged time window may also be due to a cumulative post-match daily practice by means of highly demanding activities (Banfi et al., 2012).

However, while the recovery kinetic of neuromuscular and biological parameters following a football game is well known (Ascensão et al., 2008; Nédélec et al., 2012, 2013; Draganidis et al., 2015; Romagnoli et al., 2016; Doeven et al., 2018; Silva et al., 2018; Kunz et al., 2019), only few studies have investigated the effects of different training sessions on the subsequent restoration of perceptual and biochemical variables (Andersson et al., 2008, 2010). A study compared the effects of passive versus AR (including submaximal cycling at low-intensity resistance training) between two matches separated by 72 h in elite female soccer players (Andersson et al., 2008). The AR was scheduled 22 and 46 h after the first match, and the players were tested at baseline, 0, 5, 21, 27, 45, 51, and 69 h after the first match, as well as immediately after the second match. Overall, Andersson et al. (2008) did not find significant differences at any time points on the recovery timeframe of muscle soreness and biochemical (e.g., CK) variables between active and passive recovery. Interestingly, following the first match, muscle soreness and CK values returned to baseline within 72 h (i.e., after 51 and 45 h, respectively) after both recovery interventions, which is in line with our findings. Nonetheless, Andersson et al. (2008) utilized a combination of cycling and general resistance training exercises, which do not mirror the sport-specific needs of the players and do not resemble the training practice routine usually carried out between multiple weekly games. Therefore, in the present investigation, SST was organized to meet the players’ technical, tactical, and conditioning demands during a typical pre-match session. These practices often require players to perform several explosive concentric and eccentric actions (e.g., sprints and changes of directions), which, as shown in a previous study (Trecroci et al., 2020), they seem to become somehow demanding especially for knee flexors. As such, the higher load imposed by SST compared to AR after 48 h post-match may likely have contributed to slow down the restoration of knee flexor muscle force production (Trecroci et al., 2020), thus prolonging the recovery of the related perceptual and biochemical variables up to 72 h.

The fact that SST likely elicited bigger exercise-induced muscle damage, as shown by CK changes, was also reinforced by the concurrent increase in perceived muscle soreness at 0 h. According to Nedelec et al. (2014), the number of short sprints (<5 m) performed during the match was correlated to the muscle soreness measured at both 48 and 72 h after the game. Therefore, different recovery patterns of muscle soreness scores detected between SST and AR intervention may likely be attributed to their inherent task-specific characteristics. Moreover, the fact that muscle soreness scores decreased significantly after AR would reflect a better restoration of perceptual responses linked to muscle fatigue following low-intensity training on match-day +2 (e.g., straight-line jogging).

On the contrary, no differences were observed in the time course of inflammatory (CRP and IL-6), immunological (WBC, lymphocytes, neutrophils, and monocytes), and endocrine (cortisol) markers between SST and AR, indicating that small doses of high-intensity exercise performed 2 days after the game would not seem to compromise the restoration in most of the selected biochemical variables. This is in line with Mohr et al. (2016) who, examining the effect of playing three competitive games in 1 week (with one training session between games) (Mohr et al., 2016), reported that, except for CRP (which was still high after the second/middle match), the targeted inflammatory, immunological, and endocrine markers returned to baseline within 72 h after the first match. In support, the CRP as well as lymphocyte levels from the present study did not change significantly throughout the time span and were similar in both SST and AR interventions. What emerges from the subjects’ response to the intervention is that prominent markers of inflammation and systemic stress (UA, sCr, cortisol, CRP, and IL-6) reflected the situation observed in AR for CK, i.e., a greater decrease during recovery. Although this difference did not reach statistical significance, they are possibly indicative of a better adaptation, in all subjects, consequent to the AR intervention rather than to an additional training session. However, since most of the studies examined the time course of the post-match recovery without considering the effect of the daily practice and/or specifying the drills performed during each training session, it makes it difficult to further direct comparisons with the present findings. Thus, future research is warranted to better clarify the role of daily practices on the recovery pattern of perceptual and biochemical parameters after a match or within a congested fixture period.

Overall, the results from the present study taken together with those of a previous investigation (Trecroci et al., 2020) indicate that, compared to AR, delivering high-intensity exercise 2 days after a game impairs the recovery of selected physiological (CK), perceptual (muscle soreness), and neuromuscular/mechanical components (muscle flexors MVC, soreness, and CK) but does not have a negative impact on single- and repeated-sprint capacity. Thus, it appears that the beneficial effect of post-game AR or low-intensity activities seems to be linked more to an augmented clearance of those parameters reflecting exercise-induced muscle damage, rather than to a better restoration of exercise performance and its relative biochemical variables. Of note, future research should also evaluate the changes of perceptual and biochemical responses in relation to AR and SST matched for training duration. On one hand, a shorter duration session (30 min versus 1 h) may contribute to change the training load and players’ perceptual responses, perhaps limiting data interpretation. On the other hand, employing different forms of low- to high-intensity and low- to high-duration sessions (AR and SST) may contribute to infer practical information on the recovery of perceptual and physiological markers within a realistic scenario.

The current novel findings may also be of practical relevance as they can aid to get additional knowledge in the decision-making process when prescribing specific training interventions. We cannot rule out the fact that a higher intensity or grater duration of high-intensity exercise could have led to different physiological responses on the following day. On the other hand, the current data, combined with those from Trecroci et al. (2020), seem to suggest that a low dose of high-intensity work performed 2 days after the game is not detrimental *per se* for subsequent exercise performance. However, future research is warranted to directly test this hypothesis as well as to examine its effects on both additional fitness improvements and fatigue/injury rate maintenance over a longer period. Furthermore, it should be acknowledged that the small sample size employed in the present investigation may be considered as a limitation when interpreting the study outcomes, although the utilization of a crossover design certainly strengthened the results. However, further studies will have to recruit larger sample sizes in order to confirm the findings from the current research.

## Conclusion

This study showed that the recovery pattern of match-induced perceived muscle soreness and CK perturbations was not the same when SST or AR intervention were performed 48 h after the game, with AR eliciting a quicker VAS and CK restoration compared to SST intervention at 72 h post-match. No changes in the time course recovery of inflammatory, immunological, and endocrine markers were displayed between the two interventions. Additional studies including competitive games and elite players are warranted to induce higher workload and greater ecological match-related fatigue effects. Furthermore, future research should aim at employing larger sample size for increasing the statistical power. Lastly, it would be of interest to understand how the different training strategies (low- vs. high-intensity exercise) carried out between multiple weekly matches may affect fatigue, physiological adaptations, and possible performance changes in the long term (e.g., after 6–8 weeks).

## Data Availability Statement

The raw data supporting the conclusions of this article will be made available by the authors, without undue reservation.

## Ethics Statement

The studies involving human participants were reviewed and approved by the Ethical Committee of the University of Milan. Written informed consent to participate in this study was provided by the participants’ legal guardian/next of kin.

## Author Contributions

AT, EP, and FI conceived and conducted the research. AT, EP, GL, GB, RD, and ER performed formal analysis and data curation. AT and FI wrote and drafted the manuscript. GA and FI supervised the experimental procedures. All authors have read and approved the final version of the manuscript and agreed with the order of presentation of the authors.

## Conflict of Interest

The authors declare that the research was conducted in the absence of any commercial or financial relationships that could be construed as a potential conflict of interest.
